# The developmental journey of therapies targeting purine receptors: from basic science to clinical trials

**DOI:** 10.1007/s11302-022-09896-w

**Published:** 2022-09-29

**Authors:** Seunga Han, Haruna Suzuki-Kerr, Srdjan M. Vlajkovic, Peter R. Thorne

**Affiliations:** 1 Department of Physiology, School of Medical Sciences, University of Auckland, Private Bag 92014, Auckland, 1142 New Zealand; 2 Eisdell Moore Centre, University of Auckland, Auckland, New Zealand; 3 Aotearoa Brain Project - Kaupapa Roro O Aotearoa, Dunedin, New Zealand; 4 Section of Audiology, School of Population Health, University of Auckland, Auckland, New Zealand

**Keywords:** Purinergic signalling, Therapies targeting purine receptors, Diquafosol, Prasugrel, P2X receptor, P2Y receptor, Clinical trials

## Abstract

Since the discovery of ATP as an extracellular signalling molecule in 1972, purinergic signalling, mediated by extracellular purines and pyrimidines has been identified in virtually all mammalian tissues and is implicated in regulating fundamental cellular processes. In recent years, there has been an increasing focus on the pathophysiology and potential therapeutic interventions based on purinergic signalling. A vast range of compounds targeting purine receptors are in clinical development, and many more are in preclinical studies, which highlights the fast growth in this research field. As a tribute to Professor Geoffrey Burnstock’s legacy in purinergic signalling, we present here a brief review of compounds targeting purine receptors that are in different stages of clinical trials. The review highlights the 50-year journey from basic research on purinergic receptors to clinical applications of therapies targeting purine receptors.

The concept of purinergic signalling, first proposed in 1972 [1] refers to the role of extracellular nucleosides and nucleotides, most notably adenosine and adenosine 5′-triphosphate (ATP), as signalling molecules. It was initially viewed that these molecules act as neurotransmitters, but subsequent studies extended their roles to the regulation of numerous cellular processes, including cell proliferation, differentiation, migration, and apoptosis [2]. Since 1972, four subtypes of P1 (adenosine) receptors, seven subtypes of ATP-gated ion channels (P2X receptors) and eight subtypes of P2Y G protein-coupled receptors have been cloned and sequenced [3, 4]. An increasing focus on the role of these receptors in disease and their therapeutic potential as pharmacological targets for drug development has been apparent in recent years [4, 5]. Several decades following the early pioneering work of Professor Geoffrey Burnstock, the therapeutic potential of purinergic signalling is widely recognised, and translational studies have enabled therapeutic compounds to reach clinical trials. As a tribute to Professor Geoffrey Burnstock, we have prepared this brief review of compounds now in Phase I to Phase IV clinical trials and presented several case studies to describe the translational journey of therapies targeting purine receptors to clinical practice.

Studies and reports for inclusion in this review were selected according to the criteria outlined below. An overview of therapies targeting purine receptors was provided by 110 articles containing 18 reviews, 86 original research papers, two expert opinion papers, one research abstract, one editorial paper, and two commentary papers. Comparative and randomized controlled trials, including clinical trials from Phase I to Phase IV and prospective studies, were included in this review. Three databases were used in the search: the National Institute of Health (NIH) National Library of Medicine Registry [6], the European Clinical Trials Registry [7] and the MEDLINE database (as of 5 March 2022). Key search words were P2X_1_, P2X_2_, P2X_3_, P2X_4_, P2X_5_, P2X_6_, P2X_7_ receptors, P2Y_1_, P2Y_2_, P2Y_4_, P2Y_6_, P2Y_11_, P2Y_12_, P2Y_13_, P2Y_14_ receptors, adenosine A_1_ receptor, A_2A_ receptor, A_2B_ receptor, A_3_ receptor, and the individual drug compound names. A total of 669 clinical studies were identified after the initial search. No restrictions were applied on the date of study commencement, completion, or status. The clinical trial identification number, stage of development (Phase I–IV), target disease/conditions, trial status, availability of results, statistical significance for the indicated primary outcome measure, completion of study dates, and publications associated with the trial were recorded (Tables 1, 2, 3). Where the results were published, only articles reported in English were included as references.

**Table 1:** Current registered clinical trials involving P2X receptor agonists/antagonists

Target	Compound/ Drug	Mode of Action	Condition	Latest Phase (I-IV)	Trial Identifier	Status	Date Completed	Outcome	[Ref]
P2X_3_	MK-7264/AF-219 (Gefapixant)	Antagonist	Chronic cough	II	NCT01432730	Completed	February 2013	Significant	[8]
					NCT02477709	Completed	August 2015	Significant	[6]
					NCT02502097	Completed	July 2016	Not Significant	[9]
	BAY1817080 (Eliapixant)	Antagonist	Drug interactions	I	NCT04252300	Completed	December 2020	N/A	[6]
			Chronic cough	II	NCT04562155	Completed	July 2021	N/A	[10]
			Endometriosis	II	NCT04614246	Completed	May 2022	N/A	[6]
	S-600918	Antagonist	Chronic cough	II	NCT04110054	Completed	December 2020	N/A	[6]
	BLU-5937	Antagonist	Refractive chronic cough	II	NCT04678206	Completed	November 2021	N/A	[6]
			Chronic pruritus		NCT04693195	Completed	October 2021	N/A	[6]
P2X_7_	BIL010t * (BSCT 10%)	Antibody	Basal cell carcinoma	I	NCT02587819	Completed	April 2014	N/A	[11]
	JNJ-54175446	Antagonist	Major depressive disorder, Inflammation	I	NCT03088644	Completed	November 2017	N/A	[6]
	JNJ-55308942	Antagonist		I	NCT03437590	Completed	October 2018	N/A	[6]
	CE-224,535	Antagonist	Rheumatoid arthritis	IIa	NCT00628095	Completed	February 2009	Not signficiant	[12]
	GSK1482160	Antagonist	Inflammatory pain	I	NCT00849134	Completed	April 2009	N/A	[6]

(Significant) Indicates that the study has been successfully completed and primary outcomes are statistically significant (*P* < 0.05), (Not Significant) indicates that the study has been successfully completed, but primary outcomes are not statistically significant, (N/A) indicates that the study has been completed, but results have not been published, or primary outcomes are unknown. *BLT010t is a polyclonal sheep antibody that binds to the P2X7 receptor but is not an agonist or antagonist [11]

**Table 2:** Current registered clinical trials involving P2Y receptor agonists/antagonists

Target	Compound/ Drug	Mode of Action	Condition	Latest Phase (I-IV)	Trial Identifier	Status	Date Completed	Outcome	[Ref]
P2Y_2_	Pyridoxine hydrochloride	Antagonist	Regulation of blood flow	I	NCT03738943	Completed	June 2021	N/A	[6]
	Pyridoxal-5-Phosphate	Antagonist	Regulation of blood flow	I	NCT03738943	Completed	June 2021	N/A	[6]
	Diquafosol	Agonist	Dry eye syndrome/ diabetic dry eye	IV	NCT04668118	Active	N/A	N/A	[6]
					NCT04952987	Recruiting	N/A	N/A	[6]
					NCT05193331	Not yet recruiting	N/A	N/A	[6]
P2Y_12_	Clopidogrel/ Ticagrelor/ Prasugrel + Aspirin (Acetyl salicylic acid) DAPT with/ without an underlying disease.	Antagonist	Coronary artery aneurysm, Acute coronary syndrome, cerebral aneurysm, endovascular procedures, transient ischemic attack, stroke, chronic obstructive pulmonary disease, severe aortic valve stenosis, transcatheter aortic valve implantation	IV	NCT02410083	Completed	December 2015	N/A	[6]
					NCT02049762	Completed	January 2017	N/A	[6]
					NCT02049762	Completed	January 2017	N/A	[6]
					NCT04014803	Recruiting	N/A	N/A	[6]
					NCT04483583	Recruiting	N/A	N/A	[6]
					NCT03581409	Completed	January 2021	N/A	[6]
					NCT04570345	Recruiting	N/A	N/A	[6]
					NCT02748330	Completed	December 2018	N/A	[6]
					NCT04484259	Recruiting	N/A	N/A	[6]
					NCT02224066	Completed	August 2018	Significant	[13]
					NCT01584791	Completed	February 2012	Not significant	[14]
					NCT02018055	Completed	January 2021	Significant	[15]
					NCT01830491	Completed	December 2009	Not significant	[16]
					2014-003599-21	Completed	N/A	N/A	[7]
				III	2015-005630-21	Ongoing	N/A	N/A	[7]
	Clopidogrel/ Ticagrelor/ Prasugrel (dosing/ comparative/ optimization) with/ without an underlying disease/ genotype variation.	Antagonist	Acute coronary syndrome, symptomatic aortic stenosis with/ without an underlying specific disease eg. Diabetes mellitus type II, chronic kidney disease, chronic asthma, migraine, limb ischemia, atherosclerosis, Sickle cell disease	IV	NCT01994941	Completed	August 2015	Significant	[17]
					NCT02618837	Active	N/A	N/A	[6]
					NCT02224274	Completed	June 2016	Significant	[18]
					NCT02215993	Completed	October 2014	N/A	[6]
					NCT02060786	Completed	June 2013	Significant	[19]
					NCT02064985	Completed	November 2014	Significant	[20]
					NCT02639143	Completed	December 2017	N/A	[6]
					NCT03387826	Completed	January 2019	Not significant	[21]
					NCT03774394	Recruiting	N/A	N/A	[6]
					NCT01587651	Completed	February 2013	Significant	[6]
					NCT02663713	Completed	June 2017	N/A	[21]
					2013-002734-20	Completed	October 2020	Not significant	[7]
					NCT03672097	Completed	August 2020	Significant	[22]
					NCT01603082	Completed	June 2014	Significant	[23]
					NCT01014624	Completed	June 2010	Significant	[24]
					NCT04331145	Completed	February 2022	Significant	[13]
					NCT01706510	Completed	April 2014	N/A	[6]
					NCT01305369	Completed	December 2011	N/A	[6]
					NCT01864005	Completed	March 2014	Significant	[6]
					NCT02054663	Completed	February 2015	Non-significant	[25]
					NCT02065479	Completed	April 2019	Significant	[26]
					NCT01951001	Completed	October 2018	Significant	[27]
					NCT01523366	Completed	May 2013	Significant	[6]
					NCT01523392	Completed	September 2013	Significant	[28]
					NCT02777580	Recruiting	N/A	N/A	[6]
					NCT02518464	Completed	October 2017	Not significant	[29]
					NCT03489863	Completed	March 2019	Significant	[6]
					2013-001636-22	Completed	May 2017	Significant	[30]
					NCT04001894	Completed	December 2020	N/A	[6]
					NCT02931045	Completed	December 2019	Significant	[31]
					NCT01765400	Completed	December 2015	N/A	[6]
					NCT01823510	Completed	May 2016	Significant	[32]
					NCT04654052	Recruiting	N/A	N/A	[33]
					NCT01365221	Completed	January 2014	Not significant	[34]
					NCT02287909	Completed	March 2018	Significant	[35]
					NCT02505399	Completed	May 2018	N/A	[6]
					NCT02742987	Completed	June 2015	N/A	[6]
					NCT00140465	Completed	July 2005	N/A	[6]
					NCT01950416	Completed	December 2014	N/A	[6]
					NCT01626534	Completed	February 2014	N/A	[6]
					NCT02587260	Completed	February 2017	Not significant	[36]
					NCT02026219	Completed	December 2014	N/A	[37]
					NCT04755387	Recruiting	N/A	N/A	[6]
					NCT01463163	Completed	April 2012	Not significant	[38]
					2011-003320-12	Completed	March 2013	Significant	[7]
					2012-002404-41	Ongoing	N/A	N/A	[7]
					NCT01789814	Completed	July 2014	Significant	[6]
					NCT01962428	Completed	December 2015	N/A	[6]
					NCT01538446	Completed	May 2016	Significant	[39]
					NCT01957540	Completed	December 2015	Not significant	[40]
					NCT00724880	Completed	December 2007	N/A	[6]
					NCT03462498	Active	N/A	N/A	[41]
					NCT03008083	Recruiting	N/A	N/A	[42]
					NCT02601157	Recruiting	N/A	N/A	[43]
					2012-005514-18	Completed	N/A	N/A	[7]
					2021-001418-12	Ongoing	N/A	N/A	[7]
					2011-004064-29	Completed	February 2013	Not significant	[7]
					2006-006695-38	Ongoing	N/A	N/A	[7]
					2016-004959-80	Ongoing	N/A	N/A	[7]
					2014-004238-25	Completed	November 2015	Significant	[7]
				III	2019-002391-13	Ongoing	N/A	N/A	[7]
					2020-004887-24	Ongoing	N/A	N/A	[7]
					NCT04739384	Recruiting	N/A	N/A	[44]
					NCT03357874	Recruiting	N/A	N/A	[21, 45]
					NCT03649711	Active	N/A	N/A	[21]
					NCT05077683	Recruiting	N/A	N/A	[6]
					2015-000850-39	Ongoing	N/A	N/A	[7]
				II	NCT04766437	Recruiting	N/A	N/A	[6]
					2006-002618-37	Completed	October 2007	Significant	[7]
	Clopidogrel/ Ticagrelor/ Prasugrel + opioids	Antagonist	ST-Elevation Myocardial Infarction	IV	NCT03400267	Completed	November 2019	N/A	[6]
					NCT01536964	Completed	March 2013	N/A	[6]
					NCT02217878	Completed	June 2015	Significant	[46]
					2017-002671-26	Ongoing	N/A	N/A	[7]
	Prasugrel/ Ticagrelor + Vorapaxar	Antagonist	Myocardial infarction	IV	NCT02545933	Completed	January 2020	Significant	[47]
	Clopidogrel + omeprazole and pantoprazole	Antagonist	Secondary prevention of ischemic events and coronary artery stent thrombosis.	I	NCT01170533	Completed	August 2010	Not significant	[48]
	Cangrelor	Antagonist	Acute Coronary Syndrome	I	NCT00696566	Completed	March 2008	N/A	[6]
				IV	NCT03247738	Completed	December 2018	Significant	[49]
					NCT04005729	Completed	November 2021	N/A	[6]
					NCT04634162	Completed	November 2021	N/A	[6]
					NCT04668144	Recruiting	N/A	N/A	[6]
					NCT03551964	Recruiting	N/A	N/A	[6]
					NCT02943369	Completed	December 2017	N/A	[6]
					NCT02733341	Completed	October 2018	Significant	[50]
					NCT04927949	Recruiting	N/A	N/A	[6]
					NCT02978040	Completed	December 2019	Significant	[51]
					2016-000195-19	Completed	October 2017	N/A	[7]
					2019-001285-15	Ongoing	N/A	N/A	[7]
				III	2015-005071-25	Ongoing	N/A	N/A	[7]
					2016-002586-64	Ongoing	N/A	N/A	[7]
	Selatogrel (ACT-246475)	Antagonist	Acute myocardial infarction	II	NCT03487445	Completed	November 2018	Significant	[52]
					NCT03384966	Completed	September 2018	Significant	[53]
					2018-000765-36	Completed	November 2018	N/A	[7]
					2017-003332-36	Completed	September 2018	Significant	[7]

(Significant) Indicates that the study has been successfully completed and primary outcomes are statistically significant (*P* < 0.05), (Not significant) indicates that the study has been successfully completed, but primary outcomes are not statistically significant, (N/A) indicates that the study has been completed, but results have not been published, or primary outcomes are unknown.

**Table 3:** Current registered clinical trials involving adenosine receptor agonists/antagonists

Target	Compound/ Drug	Mode of Action	Condition	Latest Phase (I-IV)	Trial Identifier	Status	Date completed	Outcome	[Ref]
A_1_	PBF-680	Antagonist	Atopic asthma	II	NCT03774290	Completed	March 2020	N/A	[6]
					NCT01939587	Completed	July 2014	N/A	[6]
					NCT02635945	Completed	November 2019	N/A	[6]
					2017-003663-35	Ongoing	N/A	N/A	[7]
	GW493838	Agonist	Peripheral neuropathic pain	II	NCT00376454	Completed	June 2003	N/A	[6]
	BAY1067197 (Neladenoson bialanate)	Agonist	Heart failure	II	NCT02040233	Completed	April 2015	N/A	[6]
					NCT03098979	Completed	June 2018	Not significant	[54]
					NCT02992288	Completed	May 2018	Not significant	[55]
					2016-003839-38	Completed	May 2018	Not significant	[7]
					2016-004062-26	Completed	June 2018	Not significant	[7]
					2013-001287-34	Completed	March 2015	N/A	[7]
					2013-002522-23	Completed	April 2015	N/A	[7]
	Tonapofylline	Antagonist	Renal insufficiency, Congestive heart failure	II/III	NCT00709865	Completed	December 2009	N/A	[6]
	DTI-0009 (Selodenoson)	Agonist	Atrial fibrillation	II	NCT00040001	Completed	N/A	N/A	[6]
	KW-3902 (rolofylline)	Antagonist	Congestive heart failure	III	NCT00354458	Completed	July 2009	Not significant	[56]
					NCT00328692	Completed	July 2009	Not significant	[56]
A_2A_	Regadenoson (CVT-3146/ Lexiscan)	Agonist	Lung transplant	I	NCT03072589	Recruiting	N/A	N/A	[57]
			Myocardial perfusion imaging	III	NCT00208312	Completed	June 2005	Significant	[58]
					NCT00208299	Completed	August 2006	N/A	[6]
			Sickle cell disease	II	NCT01788631	Completed	December 2016	Not significant	[59]
	KW-6002 (Istradefylline)	Antagonist	Parkinson’s disease	III	2004-002844-93	Completed	March 2007	Significant	[7]
					2004-000817-20	Completed	October 2005	Not significant	[7]
					2015-003887-34	Completed	December 2017	N/A	[7]
					2013-002254-70	Completed	October 2016	Significant	[7]
					2019-002951-40	Ongoing	N/A	N/A	[7]
					NCT02610231	Completed	December 2017	N/A	[6]
					NCT01968031	Completed	October 2016	N/A	[6]
					NCT00957203	Completed	March 2012	N/A	[6]
					NCT00955526	Completed	February 2011	N/A	[6]
					NCT00199420	Completed	December 2005	N/A	[6]
					NCT00199407	Completed	January 2006	N/A	[6]
					NCT00203957	Completed	February 2007	N/A	[6]
					NCT00199394	Completed	November 2005	N/A	[6]
					NCT00199368	Completed	May 2007	N/A	[6]
					NCT00955045	Completed	October 2003	N/A	[6]
	V81444	Antagonist	Parkinson’s disease	I	NCT02764892	Completed	March 2013	N/A	[6]
	PBF-509 (NIR178)	Antagonist	Non-small cell lung cancer	I/II	NCT02403193	Completed	November 2021	Significant	[60]
				II	NCT03207867	Recruiting	N/A	N/A	[6]
			Parkinson’s disease	I	NCT02111330	Completed	May 2014	N/A	[6]
					NCT01691924	Completed	October 2013	N/A	[6]
	Inupadenant	Antagonist	Antitumour activity (Advanced cancer, lung cancer, head and neck cancer, melanoma)	I/II	NCT05060432	Recruiting	N/A	N/A	[6]
	SYN115	Antagonist	Imaging in Cocaine dependence	I	NCT00783276	Completed	January 2013	Significant	[61]
	BVT.115959	Agonist	Diabetic neuropathic pain	II	NCT00452777	Completed	January 2008	N/A	[6]
	Ciforadenant	Antagonist	Renal cell cancer, metastatic castration resistant prostate cancer	I	NCT02655822	Completed	July 2021	N/A	[6]
	Radiotracer [18F]MNI-444*	Radio tracer for positron emission tomography (PET)	Parkinson’s disease imaging	I	NCT05009199	Recruiting	N/A	N/A	[62]
	Marker [123]MNI-420*	Radio tracer for PET	Parkinson’s disease and Huntington’s disease imaging	I	NCT00970229	Completed	May 2015	N/A	[63]
A_2B_	PBF-1129	Antagonist	Locally advanced or metastatic non-small cell lung cancer	I	NCT03274479	Recruiting	N/A	N/A	[6]
A_3_	PBF-1650	Antagonist	Psoriasis	I	NCT03798236	Completed	May 2019	N/A	[6]
	PBF-677	Antagonist	Glaucoma	I	NCT02639975	Completed	June 2016	N/A	[6]
			Ulcerative colitis	II	NCT03773952	Completed	May 2021	N/A	[6]
	CF101	Agonist	Plaque psoriasis	III	NCT03168256	Active, not recruiting	N/A	N/A	[6]
			Rheumatoid arthritis	III	2016-003682-26	Ongoing	N/A	N/A	[7]
	CF102	Agonist	Advanced Hepatocellular Carcinoma	II	NCT02128958	Completed	December 2021	Not signficant	[64]
			Advanced Hepatocellular Carcinoma with Child-Pugh Class B Cirrhosis	II	2014-000489-23	Completed	March 2019	Not significant	[7]
			Chronic hepatitis C	I/II	NCT00790673	Completed	July 2011	N/A	[6]

(Significant) Indicates that the study has been successfully completed and primary outcomes are statistically significant (*P* < 0.05), (Not significant) indicates that the study has been successfully completed, but primary outcomes are not statistically significant, (N/A) indicates that the study has been completed, but results have not been published, or primary outcomes are unknown. *[18F] MNI-444 and [123]MNI-420 are selective A_*2A*_* receptor radiotracers used to study neurodegenerative and neuropsychiatric disorders *in vivo* to support drug-discovery studies targeting A*_*2A*_* receptors *[62, 63]

## Compounds in clinical trials

**Fig. 1:**
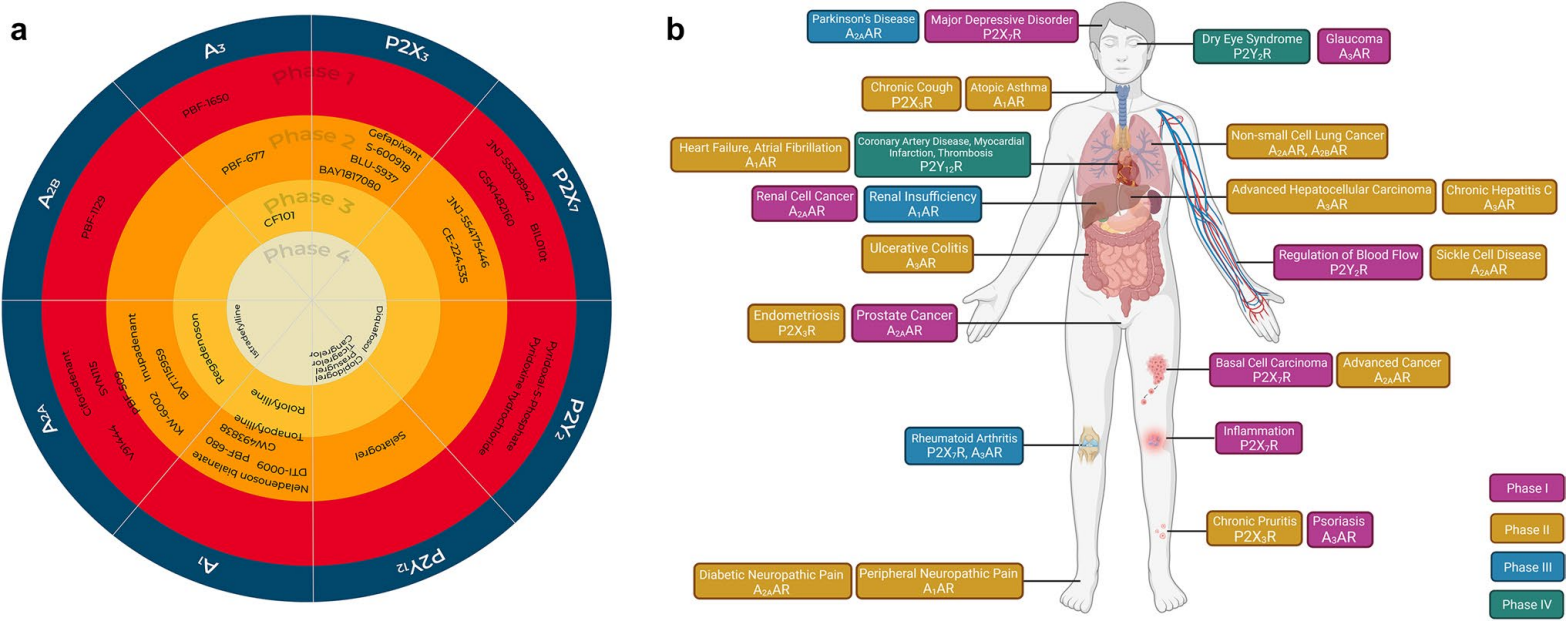
Summary of therapeutic compounds in clinical trials targeting purine receptors for specific diseases/conditions. (**A**) Therapeutic compounds targeting purine receptors in clinical development from Phase I–IV. (**B**) Purine receptors targeted for specific diseases/conditions in clinical development from Phase I–IV, created with BioRender.com

Data from the clinical registries showed that 38 compounds, all agonists and antagonists targeting purine receptors, are currently in clinical development (Tables 1, 2, 3). Four compounds target the P2X_3_ receptor, and five the P2X_7_ receptor (Table 1). For P2Y receptors, three compounds target the P2Y_2_ receptor and different generations of the same compound target the P2Y_12_ receptor (Table 2). Six compounds target the A_1_ receptor, ten compounds the A_2A_ receptor, one the A_2B_ receptor_,_ and one the A_3_ receptor (Table 3, Fig. 1A). These compounds are in different stages of clinical trial. In Phase I, eleven compounds target P2X_7_, P2Y_2_, A_2A_, and A_2B_ receptors. Seventeen compounds targeting P2X_3_, P2X_7_, P2Y_12_, A_1_, A_2A_, and A_3_ receptors are in Phase II. Only three compounds are in Phase III, targeting A_1_, A_2A_, and A_3_ receptors. Six ligands targeting P2Y_2_, P2Y_12_ and A_2A_ receptors are in Phase IV under post-approval surveillance evaluation (Fig. 1A). The greater number of compounds targeting the A_2A_ and P2X_7_ receptors highlights their potential in pharmacotherapy (Table 3). The A_2A_ and P2X_7_ receptors have gained more attention, likely due to their involvement in inflammatory and immune processes [55]. For example, A_2A_ receptor agonists can suppress peripheral inflammation, and antagonists are used as adjuvant treatments for neuroinflammatory diseases [54]. P2X_3_, P2X_4_, P2X_7_ receptors have been implicated in signalling pathways for pathological pain [65, 66]. In particular, activation of P2X_3_ subtype expressed in the central terminals of dorsal root ganglia increases nociception by sensitizing nerve fibers associated with the transmission of pain [65]. Blocking P2X_3_ activities by selective P2X_3_ antagonists shows promise in reducing pain associated with inflammatory, neuropathic, chronic, and cancer-induced conditions in preclinical studies [65]. P2X_7_ receptors have also gained attention due to their role in regulating inflammation and the innate and adaptive immune responses [67]. Many studies have focused on manipulating the relation between P2X_7_ receptor activation and subsequent release of inflammatory cytokines, which can promote cell proliferation or apoptotic cell death [55]. For example, the P2X_7_ receptor is implicated as a mediator of cancer invasion and metastasis; hence, it has been investigated as a target to inhibit cancer progression [11, 55]. As inflammation and immune processes are involved in most pathologies, sixteen different conditions were identified as targets for purine receptor-based therapies with P1 or P2 receptor ligands under clinical trial (Fig. 1B). Advanced cancer and complications associated with acute coronary syndrome (ACS) and/or cardiovascular disease (CD) appear to be the most targeted, with five different compounds in development for each condition. However, not all compounds were successful in providing significant clinical outcomes. It is not uncommon for compounds to result in less than optimal clinical efficacy when tested in humans despite displaying promising efficacy in preclinical animal studies [66]. For example, P2X_7_ subtype-specific antagonist AZD9056 showed promising results in an in vivo rat model by suppressing symptoms of rheumatoid arthritis [68, 69]. However, AZD9056 failed to show efficacy for rheumatoid arthritis in a Phase IIa study (NCT00520572). Another unsuccessful example is Rolofylline (KW-3902), an A_1_ receptor antagonist investigated for preventing renal dysfunction. Although Rolofylline appeared to show potential benefits in improving kidney function in patients with congestive heart failure in a Phase II trial (NCT00159614) [70], it failed to show efficacy in Phase III clinical trials (NCT00354458, NCT00328692) [56] (Table 3). Although there is no definitive explanation for why some trials fail to show clinical efficacy, factors that may influence outcomes include inter-species differences in receptor function or pharmacology, variation in pharmacological profile of compounds for homomeric and heteromeric receptor complexes, and inter-individual variation in receptor function due to single nucleotide polymorphisms [65, 66]. Furthermore, the ubiquitous nature of purinergic receptor expression throughout the body can potentially increase the risk of side effects that may outweigh clinical benefits [71]. However, there are many examples of successful drug development. BIL010t (formerly known as BSCT), a non-functional form of the P2X_7_ receptor (nf-P2X_7_) antibody used as topical therapy for basal cell carcinoma [11], and PBF-509, a potent A_2A_ receptor antagonist for the treatment of non-small cell lung cancer [60], were both well-tolerated and safe in Phase I/II trials [11] (Table 3).

The development of therapeutics generally follows an exhaustive translational research path from discovery to clinical trial. Approximately 38 compounds targeting purine receptors are at different stages of development, and the most extensively studied are the P2Y_12_ receptor-based antiplatelet agents clopidogrel, ticagrelor and prasugrel. These are all approved for treating complications associated with the ACS as percutaneous coronary intervention (PCI) [72]. Diquafosol (previously known as INS365), a P2Y_2_ receptor agonist, is another approved drug that has been commercially successful in treating dry eye syndrome [73]. To illustrate the translational journey from basic science to clinical trials, we present case studies of diquafosol, and a family of P2Y_12_ receptor antagonists.

## P2Y_2_ receptor agonist: diquafosol

Diquafosol (Tetrasodium ophthalmic solution 3%; INS365, Prolacria, DIQUAS®) is a synthetic dinucleotide derivative of naturally occurring uridine 5′-triphosphate (UTP), acting as a potent P2Y_2_ receptor agonist [74]. Diquafosol targets P2Y_2_ receptors expressed in ocular tissues, including palpebral and bulbar conjunctival epithelium, conjunctival goblet cells, corneal epithelial, and meibomian glands [74]. It is used to treat dry eye syndrome by stimulating tear fluid secretion from conjunctival goblet cells, promoting ocular surface hydration and stabilization of the tear film, independent of tear fluid secretion from lacrimal glands [73].

For details on the current use of diquafosol and its tolerance by patients, the reader is directed to a review by Keating et al., 2015 [75]. Here, we will focus on the discovery and translational journey. Basic science discoveries for the target receptor of diquafosol, P2Y_2_, were published in early 2000–2003. Early work identified P2Y_2_ receptor mRNA expression to be highly conserved across species, from rhesus monkeys to white rabbits, with expression observed in the conjunctiva, cornea, ciliary body, lens, and pigmented epithelium [76]. Pharmacological and functional evidence of diquafosol action on P2Y_2_ receptors has stemmed from several cell line and animal models, including non-transformed bovine ciliary epithelial cells [77], human non-pigmented ciliary epithelial cells [78], rabbit ciliary epithelial cells [79], and dry-eye rat model [74]. Earlier in 1999, diquafosol was developed as a P2Y_2_-specific agonist INS365 (note, the name subsequently changed to diquafosol), effective in airway clearance in sheep [80]. At that time, Inspire Pharmaceuticals had already announced its potential use for dry eye syndrome [81]. The effect of diquafosol on ocular tissues for dry eye treatment was published in 2001 [74]. These preclinical studies were important in demonstrating the mechanism of action of P2Y_2_ receptor agonists on eye physiology. For example, diquafosol administration in rabbit conjunctiva led to stimulation of Cl^−^ and water transport from the serosal to mucosal conjunctival epithelium [82]. The observed increase in tear fluid secretion was dependent on the dose of diquafosol with no significant change in tear fluid composition [82, 83]. From a clinical viewpoint, a therapeutic effect of diquafosol was demonstrated using the rat dry-eye model, as the application of diquafosol (3.0% or 8.5%) resulted in a 1.5-fold transient increase in tear fluid secretion accompanied by a decline in corneal permeability (51% compared to controls). Both effects resulted in an overall improvement in ocular surface hydration [74]. Additionally, diquafosol improved ocular surface health and mitigated corneal epithelial damage caused by superficial punctate keratopathy. An instillation of diquafosol was associated with an increase in Periodic acid-Schiff (PAS)-stained positive goblet cells in the rat dry-eye model [74] and a significant decrease in corneal fluorescein staining scores compared to controls in a diabetic rat model [84].

Following substantial research and publications between 2000 and 2003, diquafosol was granted priority in the review process by the FDA in 2003 as a diquafosol tetrasodium ophthalmic formulation [85, 86], driving a cascade of clinical trials. The translation of the potential therapeutic effects observed in the preclinical studies can be illustrated by two pivotal Phase IIb and Phase III clinical trials. Both were randomized, double-blind, multi-centre trials, with a primary outcome measure defined as improving corneal and conjunctival epithelial damage after 4 weeks [87, 88]. In the Phase IIb (NCT01189032) study involving 286 participants, a greater dose-dependent reduction in fluorescein corneal staining scores in week 4 was reported in patients receiving either 1% or 3% diquafosol (DQS) [87]. However, when examining the maintenance of fluorescein corneal staining scores at week 6, only the 3% DQS group scores were significantly different from placebo controls (*P* = 0.005) [87]. Both 1% and 3% DQS groups showed a decrease in fluorescein corneal staining scores (1% DQS, *P* = 0.037; 3% DQS, *P* = 0.002) and rose bengal corneal staining scores (1% DQS, *P* = 0.007; 3% DQS, *P* = 0.003) compared to placebo at week 4 [87]. This study included subjective dry eye sensation symptom scores as a secondary outcome measure. Patients receiving DQS showed a significant improvement in this secondary outcome measure compared with the placebo (1% DQS, *P* = 0.003; 3% DQS, *P* = 0.033) [87]. Concerning dosage, 3% DQS was superior to 1% DQS in efficacy [87]. The efficacy of 3% DQS was also superior to 0.1% sodium hyaluronate ophthalmic solution [89]. Similar to the Phase IIb study, both fluorescein and rose bengal corneal staining scores showed significant improvement in both treatment groups at weeks 2 and 4 (*P* < 0.05), although the 3% DQS treatment group exhibited greater improvement in mucin coating on the ocular surface at week 4 than 0.1% sodium hyaluronate [89]. Similar improvement in subjective dry-eye-related symptoms and corneal and conjunctival fluorescein staining scores was also observed in a larger Phase III study involving 3196 patients with dry eye disease conducted over two months (*P* < 0.001) [88]. Adverse reactions to 3% DQS treatment affected 6.3% of patients, including eye discharge, eye irritation, and eye pain [88]. Additional information regarding the effectiveness of 3% DQS as an intervention for dry-eye syndrome was delineated in a Phase IV study involving 580 patients with dry eye disease [90]. It was a prospective, multi-centre, open-label observational study conducted over 12 months [90]. Significant improvements in fluorescein corneal staining scores were observed at 3, 6, 9 and 12 months with DQS treatment (*P* < 0.001) [388]. Moreover, DQS treatment was associated with a significant reduction in other outcome measures such as Dry Eye-related Duality of Life Scores, ocular symptoms scores, and impact on daily life scores (*P* < 0.001) [90]. However, it should be noted that the open-label design of this study can be subject to bias in favor of DQS treatment in efficacy measures [90]. The study population included a higher proportion of elderly patients (42.8%, ≥ 70 years), which should also be taken into consideration [90]. DQS 3.0% ophthalmic solution was approved as an intervention for dry eye in 2010 by the Ministry of Health in Japan [91] and is now widely available in other countries, including South Korea, Indonesia, Malaysia, Philippines, Thailand, Vietnam, Cambodia and China [92]. The contributing factors to the success of diquafosol can be attributed to its localized direct delivery in the form of an ‘eye-drop’ and ease of assessment of the target tissue.

## P2Y_12_ receptor antagonist: thienopyridines prasugrel and cangrelor

The discovery of P2Y_12_ as the drug target for coronary diseases dates back to 1961, when ADP was found to play a functional role in platelet activation and aggregation [93]. The breakthrough came in 2001 when the P2Y_12_ receptor was cloned and recognized as the molecular identity of the receptor responsible for triggering the potent ADP-induced antithrombotic activity [94]. Strikingly, the expression of P2Y_12_ was found to be very specific to platelets and showed negligible expression in most other tissues [94]. The strong link between the P2Y_12_ and ADP-induced platelet aggregation has set a rapid rise in investigations of P2Y_12_ as a potential therapeutic target for blood clotting conditions and led to the development of thienopyridine prodrugs [95, 96]. Thienopyridines are a family of closely related prodrugs. As prodrugs, thienopyridines need to be metabolized by a hepatic enzyme, Cytochrome P-450 (CYP), into the active metabolite to bind to the P2Y12 receptor irreversibly [97]. This blocks ADP binding, subsequently inhibiting platelet activation and aggregation [97]. The actions of thienopyridines are specific to P2Y_12_ receptor and selectively interfere with platelet activation and aggregation induced by ADP [98, 99].

Subsequent research has led to the development of at least three thienopyridine compounds in the market; first-generation ticlopidine (Ticlid ®), second-generation clopidogrel bisulfate (Plavix®) and third-generation prasugrel (Effient®) [100, 101].

Thienopyridine antiplatelet drugs are indicated for managing and preventing complications after ACS and PCI, including ischemic complications, myocardial infarction (MI), and stent thrombosis [102], albeit with slightly different properties. The first generation ticlopidine was replaced by the second generation clopidogrel due to a better tolerability profile but with similar efficacy. Soon dual antiplatelet therapy with aspirin and clopidogrel became the ‘gold standard’ for patients undergoing stenting and acute coronary syndromes [100, 103]. However, reports of inter-individual variability in responsiveness to clopidogrel from in vitro studies sparked concerns, potentially explained by genetic polymorphisms and cytochrome P-450 polymorphisms, which can manifest as differential pharmacodynamic and therapeutic responses [104]. A heightened platelet reactivity (thienopyridine hypersensitivity reaction) or clopidogrel non-responsiveness (also referred to as clopidogrel resistance) was associated with a high risk of adverse ischemic events such as stent thrombosis [100, 105]. This supported the development of the third-generation thienopyridine prasugrel, which exhibited a superior pharmacodynamic profile to clopidogrel with less interpatient variability and a more potent platelet aggregation response [100, 103, 105]. Pharmacological and functional evidence of prasugrel (CS-747, LY640315, Effient®), 5-[2-cyclopropyl-1-pyridin-2yl acetate] and P2Y_12_ receptors have been attributed to preclinical research across a number of animal models including rats, beagle dogs, and cynomolgus monkeys [95, 106, 107]. One of the first studies to evaluate the therapeutic effect of prasugrel (CS-747) used a rat model, where single oral administration of prasugrel (0.3–3 mg/kg) produced a dose-related inhibition of ex vivo ADP-induced aggregation in washed platelets [95]. The same dose-dependent inhibition of platelet aggregation following oral administration of prasugrel was observed in a rat model where maximum inhibition was achieved 2–4 h after dosing [106]. A similar potent, dose-related inhibition of ADP-induced platelet aggregation was observed in beagle dogs (0.03–3 mg/kg/day), cynomolgus monkeys (0.1–0.3 mg/kg/day), and rats (3 mg/kg/day) across a 14-day treatment period. Inhibition reached a plateau on days 3 and 5, suggesting a cumulative effect [107]. The order of potency (from high to low) in different animal models was: dogs, humans, monkeys, and rats [107]. When comparing the antiplatelet and antithrombotic potency of prasugrel with its predecessors, the potency of prasugrel exceeded that of clopidogrel and ticlopidine in a rat arterio-venous shunt model [95]. In an ex vivo rat model, clopidogrel exhibited a slower onset of action and antiplatelet potency 13 times lower than prasugrel [106]. A dose-dependency study of prasugrel (0.1–1 mg/kg/day, p.o.) in a rat carotid arterial thrombosis model demonstrated dose-related prolongation of the time to arterial occlusion. CS-747 (prasugrel) had approximately tenfold and 100-fold higher potency when compared to clopidogrel (1–20 mg/kg/day, p.o.) and ticlopidine (30–300 mg/kg/day, p.o.), respectively [107].

The progress of prasugrel from the preclinical studies to clinical trials can be followed from the Phase II JUMBO-TIMI 26 study [108], which served as a feasibility study for one of the pivotal Phase III studies, TRITON-TIMI 38 [109]. The JUMBO-TIMI 26 study involved 904 patients and was the first to report on the use of prasugrel in patients undergoing elective or urgent PCI [97]. Patients were randomized into 1 of 3 prasugrel dosing regimens: low dose (40 mg loading dose (LD) and 7.5 mg maintenance dose (MD)), intermediate-dose (60 mg LD and 10 mg MD), and a high-dose (60 mg LD and 10 mg MD). A control group treated with clopidogrel (300 mg LD and 75 mg MD) was also included [108]. Although patients receiving prasugrel had a slightly lower incidence of major adverse cardiac events, including myocardial infarction, stroke, recurrent myocardial ischemia requiring hospitalization, and thrombosis (7.2%) compared to patients receiving clopidogrel (9.4%), the difference was not statistically significant (*P* = 0.26) [108]. Patients receiving prasugrel had slightly lower rates (0.5%) of major bleeding, significant bleeding and transfusion events compared to the clopidogrel group (0.8%), although it also was not statistically significant (*P* = 0.590) [108]. TRITON-TIMI 38 (NCT00097591) was a double-blind, double-dummy, parallel-group, multi-centre, multinational Phase III clinical trial [109]. It involved 13,608 subjects with moderate- to high-risk ACS with planned PCI who were randomized to receive either clopidogrel (300 mg LD and 75 mg MD) or prasugrel (60 mg LD and 10 mg MD) daily for 6–15 months [109]. The primary outcome measure was defined as a combination of cardiovascular death, non-fatal MI, or urgent target vessel revascularization at 30 days [109]. Patients receiving prasugrel reported fewer cardiovascular events (primary outcome measure composite) of 9.9% compared to 12.1% reported from patients receiving clopidogrel (Hazard Ratio (HR), 0.81; 95% Confidence Interval (CI), 0.73–0.090; *P* < 0.001) [109]. This was driven by a significant reduction in ischemic events among patients receiving prasugrel including myocardial infarction (9.7% for clopidogrel vs. 7.4% for prasugrel; *P* < 0.001), urgent target vessel revascularization (3.7% for clopidogrel vs. 7.4% for prasugrel; *P* < 0.001), and stent thrombosis (2.4% clopidogrel versus 1.1% prasugrel; *P* < 0.001), but there was no significant difference in the rate of stroke [109]. However, prasugrel treatment was associated with an increase in the rate of major bleeding (2.4% prasugrel vs. 1.8% clopidogrel; HR, 1.32; 95% CI, 1.03–1.68; *P* = 0.03) and life-threatening bleeding (1.4% vs. 0.9%; *P* = 0.01) events, inclusive of nonfatal bleeding (1.1% vs. 0.9%; HR, 1.25; *P* = 0.23) and fatal bleeding (0.4% vs. 0.1%; *P* = 0.002) [109]. There was no significant difference in overall mortality between treatment groups [109]. Notably, in a different Phase III study, TRILOGY-ACS trial (NCT00699998), the more serious or life-threatening bleeding events observed with the prasugrel group in the TRITON study were not observed [110]. This particular trial had a long follow-up of up to 2.5 years, and the risk of the major bleeding event was observed to be similarly low in the two treatment groups, prasugrel (10 mg daily) and clopidogrel (75 mg daily) [110, 111]. As for the efficacy, at 30 months, no significant difference was observed in the rate of death from cardiovascular causes, MI or stroke among patients under the age of 75 (13.9% in the prasugrel group and 16% in the clopidogrel group; HR 0.91; 95% CI 0.79–1.05; *P* = 0.21) [110]. However, a lower risk of multiple recurrent ischemic events was observed among patients under the age of 75 receiving prasugrel (6%) compared to the clopidogrel group (13%) after 12 months of treatment (HR 0.94; 95% CI 0.79–0.86; *P* = 0.018), consistent with findings from the TRITON study [109, 110]. In July 2009, prasugrel gained FDA approval as an intervention for reducing thrombotic cardiovascular events in patients with ACS managed with PCI, with a warning indicating a higher risk of bleeding events [112]. Several years after prasugrel became available for clinical use, some concerns were raised in subsequent analysis from the TRILOGY-ACS trial regarding the long-term effects of prasugrel and clopidogrel [113]. In 2020, the manufacturer, Eli Lilly and Co., discontinued production of prasugrel as a business decision; hence it is no longer available in Canada [114] and New Zealand [115]. After many years of success, the gap left by the withdrawal of prasugrel further prompted the use and investigation of reversible non-thienopyridine agents ticagrelor and, most recently, cangrelor.

Cangrelor (also known as AR-C69931MX) is a non-thienopyridine ATP analogue that has different actions to thienopyridines, and is a class of selective antagonists of the P2Y_12_ receptor [116]. Similar to thienopyridine, cangrelor is a direct-acting antagonist of the P2Y_12_ receptor; however, unlike thienopyridines, the inhibitory action of cangrelor is reversible [116]. It is delivered intravenously and is characterized by a rapid onset of action with a fast offset of effects due to its short plasma half-life of 3 to 6 min [116]. Furthermore, due to the drug’s short half-life, the platelet function returns to normal within 30 to 60 min after intravenous infusion of Cangrelor [116]. This fast termination of action makes Cangrelor an attractive compound by reducing the risk of potential ischemic or thrombotic complications [116–118]. In a Phase II BRIDGE trial (Bridging Anti-Platelet Therapy With Intravenous Agent Cangrelor In Patients Undergoing Cardiac Surgery, NCT00767507), a greater proportion of patients with ACS treated with Cangrelor (0.75 μg/kg per minute) had low levels of platelet reactivity during the treatment period compared with placebo (*P* < 0.001), with a low risk of thrombotic events [117]. However, in a large-scale international Phase III trial, CHAMPION (Cangrelor Versus Standard Therapy to Achieve Optimal Management of Platelet Inhibition) PCI trial (NCT00305162) reported a contradictory outcome that cangrelor (bolus of 30 μg/kg plus an infusion at 4 μg/kg per minute) was not superior to clopidogrel (loading dose of 600 mg) in reducing the primary composite death, myocardial infarction, or ischemia-driven revascularization at 48 h (*P* = 0.59) in patients with ACS before PCI [118]. This study was terminated due to insufficient evidence of the clinical effectiveness of cangrelor [6]. Another separate Phase III trial, CHAMPION PHOENIX (NCT01156571) reported that cangrelor significantly reduced the rate of myocardial infarction, ischemia-driven revascularization, or stent thrombosis at 48 h (adjusted odds ratio [OR] with Cangrelor, 0.78; 95% CI, 0.66 to 0.93; *P* = 0.005) with a lower rate of stent thrombosis compared to clopidogrel (OR, 0.62; 95% CI, 0.43 to 0.90; *P* = 0.01) [119]. Although several ongoing studies will likely provide additional insight into the clinical use of cangrelor (Table 2), further evaluations on the safety and efficacy of cangrelor are needed.

## Conclusion

The development of therapies targeting purine receptors has been a long journey that began with basic research characterising P1 and P2 receptors in health and disease, and this led to the identification of potential therapeutic targets for different conditions. Through the insights gained from preclinical studies and with the increasing interest from clinical researchers, the development of therapies targeting purine receptors became a reality. With 38 therapeutic compounds currently in clinical trials, we should expect more to emerge over the next few decades. Furthermore, building on our knowledge of purinergic signalling in various tissues and the existence of many drug candidates in the pipeline, repurposing the existing drugs as alternative pathways for drug development should also be considered. Substantial progress made in the last two decades is a true reflection of Professor Geoffrey Burnstock’s legacy that has established the field of purinergic signalling and paved the way for the development of purine receptor targeting therapies for several diseases and clinical conditions.

## Data Availability

Clinical trial information included in this publication are available in a public repository specified in the manuscript.
